# Self-alignment of silver nanoparticles in highly ordered 2D arrays

**DOI:** 10.1186/s11671-015-0804-8

**Published:** 2015-03-01

**Authors:** Ericka Rodríguez-León, Ramón Íñiguez-Palomares, Efraín Urrutia-Bañuelos, Ronaldo Herrera-Urbina, Judith Tánori, Amir Maldonado

**Affiliations:** Departamento de Física, Universidad de Sonora, 83000 Hermosillo, Sonora México; Departamento de Investigación en Física, Universidad de Sonora, 83000 Hermosillo, Sonora México; Departamento de Ingeniería Química y Metalurgia, Universidad de Sonora, 83000 Hermosillo, Sonora México; Departamento de Investigación en Polímeros y Materiales, Universidad de Sonora, 83000 Hermosillo, Sonora México

**Keywords:** Silver nanoparticles, Non-aqueous microemulsions, Patterned materials, Electron microscopy

## Abstract

We have synthesized silver nanoparticles in the non-polar phase of non-aqueous microemulsions. The nanocrystals have been grown by reducing silver ions in the microemulsion cylindrical micelles formed by the reducing agent (ethylene glycol). By a careful deposit of the microemulsion phase on a substrate, the micelles align in a hexagonal geometry, thus forming a 2D array of parallel strings of individual silver nanoparticles on the substrate. The microemulsions are the ternary system of anionic surfactant, non-polar solvent (isooctane), and solvent polar (ethylene glycol); the size of synthesized nanoparticles is about 7 nm and they are monodisperse. The study of the microstructure was realized by transmission electron microscopy, high-resolution technique transmission electron microscopy (HR-TEM), and Fourier processing using the software Digital Micrograph for the determination of the crystalline structure of the HR-TEM images of the nanocrystals; chemical composition was determined using the energy-dispersive X-ray spectroscopy. Addition technique polarizing light microscopy allowed the observation of the hexagonal phase of the system. This method of synthesis and self-alignment could be useful for the preparation of patterned materials at the nanometer scale.

**Electronic supplementary material:**

The online version of this article (doi:10.1186/s11671-015-0804-8) contains supplementary material, which is available to authorized users.

## Background

One of the main goals of nanotechnology is to devise methods for preparing nanoparticles of different materials as well as structured arrays at the nanoscale [1-23]. One-, two-, and three-dimensional arrays have applications in electronics, photonics, biodiagnosis, chemical sensing, among other fields. When dealing with nanoparticles, some of the desired nanostructures are monolayers, linear strings, crystal arrays, branched patterns, etc. Some of these arrays can be obtained by methods such as biomolecular nanolithography [2], scanning probe microscopy lithography coupled with surface modification of the substrate [3,4], magnetic sorting [5], dewetting [6,24], or drying of liquid droplets [7,25]; in some cases, the particles lie inside nanostructures such as those formed by block copolymers [8-11] or a polymer thin film matrix [12,13]. In this work, we show that it is possible to obtain linear arrays of metallic nanoparticles by nucleating them inside the cylindrical micelles of microemulsions and then depositing the sample on a substrate. The arrays are composed of parallel lines of nanoparticles and extend over distances in the micrometer scale. These arrangements could be useful in applications such as photonics [26,27], electronics [28,29], plasmonics [12,22,23,29], and biosensing [26]. The main interests of aligned nanoparticles are generation of an environment ordered to enhance the optical properties (optical response) of the system and to enhance the electrical conductivity and use of these features to applications like solar cells to obtain green energy.

For these applications, it is of current interest the synthesis of metal, dielectric, and semiconductor nanoparticles of controllable size and shape. One further step is the development of experimental procedures that allow the formation of nanostructured patterns with the particles. In our method, we nucleate silver nanocrystals from Ag^+^ ions provided by silver nitrate (AgNO_3_) inside microemulsions. The nanoparticle arrays are obtained when the samples are spread and allowed to dry over a substrate. The Ag^+^ ions are reduced to Ag atoms by ethylene glycol, the polar phase in the microemulsion. A related procedure has proved successful in the synthesis of spherical silver nanoparticles in solutions of AgNO_3_ in ethylene glycol [30]. The main difference with our approach is that we confine the Ag^+^ ions in narrow cylindrical channels, which are obtained by microemulsifying ethylene glycol with isooctane; the microemulsions have been stabilized with an anionic surfactant: dioctyl sodium sulfosuccinate (AOT).

## Methods

AgNO_3_ (99%), AOT (≥99%), ethylene glycol (EG) (99%), isooctane (IC) (99%), and AgNO_3_ were purchased in Sigma-Aldrich (Toluca, México). The chemicals were used as received.

The microemulsions were prepared by mixing appropriate amounts of isooctane, AOT, and ethylene glycol. AOT was first dissolved in isooctane; then ethylene glycol was added and the system was vigorously mixed and homogenized in a vortex. The sample was kept at ambient temperature several days for equilibration. The molar proportion of each component in the microemulsion where most of the experiments were performed was 5.8% (ethylene glycol), 90.1% (isooctane), and 4.1% (AOT). Similar results were obtained in other compositions (in the Additional file 1: Figure S1). The micelle volume fraction was calculated as the ratio of the micelle (ethylene glycol + AOT) volume to the total volume of the sample. The experimental value, as calculated from the sample composition for the used microemulsion, is ϕ_mic_ = 0.1147.

The silver nanoparticles were synthesized by adding AgNO_3_ to the microemulsions; the AgNO_3_ concentration in the sample was 20 mM in most of the experiments. In some experiments, the AgNO3 concentration was 60 mM. The sample was stirred for homogenization. The reaction was qualitatively monitored by visual inspection; we obtain photographs that show the color change when the synthesis is finished. UV-vis spectroscopy was used in order to assess the presence of silver nanocrystals. The particles were further characterized by transmission electron microscopy (TEM, both conventional and high resolution). A drop of the nanoparticle-containing microemulsion was deposited in a TEM grid; the excess solvent and surfactant was eliminated by carefully washing the grid several times with isooctane. The grid was vacuum-dried at room temperature 72 h prior to TEM observation. We have analyzed the samples in a JEOL 2010 F TEM (JEOL, México, D.F., México) operating at 200 kV. The nanoparticle diameter was measured directly from the TEM pictures with image treatment software. The polydispersity index was calculated as $\varepsilon \equiv \frac{\sigma }{D}$, where *σ*^2^ = 〈*D*^2^〉 − 〈*D*〉^2^, 〈*D*〉 being the average diameter of the particles.

## Results and discussion

**Figure 1:**
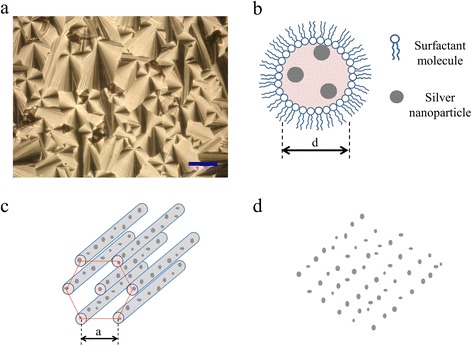
**Silver nanoparticle formation. (a)** Polarizing optical microscopy, the scale bar indicates 100 μm. **(b)** Cross section of a cylindrical micelle. **(c)** Depiction of the hexagonal array. **(d)** Expected nanoparticle array.

The microemulsions are prepared with ethylene glycol, the reducing agent in the synthesis, isooctane, and AOT. Since ethylene glycol is a polar solvent, it does not mix with isooctane, an organic liquid. However, when a surfactant, like AOT, is added to the system, it places itself in the polar-organic phase boundary due to its amphiphilic nature. In this way, ethylene glycol micelles can be suspended in isooctane, thus forming a microemulsion. The surfactant concentration, [AOT], controls the shape of the aggregates; at first, they are spherical, but upon increasing [AOT], the micelles become cylindrical and eventually they percolate in a connected network [31]. This percolation can be followed through transport experiments such as electrical conductivity measurements [32]. We have explored the isooctane-rich corner of the microemulsion phase diagram, and we have measured the electrical conductivity (*κ*) along a dilution line (a line where [AOT] increases, leaving the ethylene glycol concentration fixed). For this measure, we use the CDM210 Conductivity Meter (Radiometer, Westlake, OH, USA). We measure electrical conductivity of samples with different concentrations of AOT and isooctane where the ethylene glycol is constant, and the pole-conductivity cells are introduced into the sample then the conductivity was obtained by generation of the electric field in the pole forming by two parallel metallic slides; the equipment has a wide range from 0.01 μS/cm to 400 mS/cm using a cell constant of 1 cm − 1. All measurements were realized to room temperature. We have found a regular increase in *κ* as the surfactant concentration is increased (in the Additional file 1: Figure S2), an effect indicating a shape transformation of the aggregates (from spheres to cylinders) and perhaps some degree of percolation. These results are similar to those reported in the literature [31]. At a defined value of [AOT], *κ* reaches a maximum and decreases if [AOT] is further increased. We have chosen this high-conductivity microemulsion region as the reacting system for our synthesis because its local structure is that of cylindrical aggregates. This is confirmed by the cone-like birefringent texture observed with polarizing optical microscopy when our phase is allowed to dry on a substrate. These cone-like textures are characteristics of an hexagonal geometry. In Figure 1a, we present a polarizing optical microscopy image for the mesophase of the microemulsion with silver nanoparticles synthesized inside of the mesophase. The sample was deposited over a microscope slide and dewetting was conducted to room temperature (25°C). Image was obtained 24 h after deposition using an Olympus inverted microscope, model IX71 (Olympus Corporation, México, D.F., México). The hexagonal geometry (cone-like birefringent textures) of the microemulsion is kept when silver nanoparticles are present on the mesophase. In fact, the observed textures correspond to phases of cylindrical micelles packed in a hexagonal symmetry [33] (Figure 1b,c); the nanoparticles grow inside the cylinders, in ethylene glycol; and isooctane is outside the cylinders. We anticipate that the silver nanoparticles nucleated inside these channels will maintain the geometry and align along patterns such as that depicted in Figure 1d, when deposited on a substrate.

**Figure 2:**
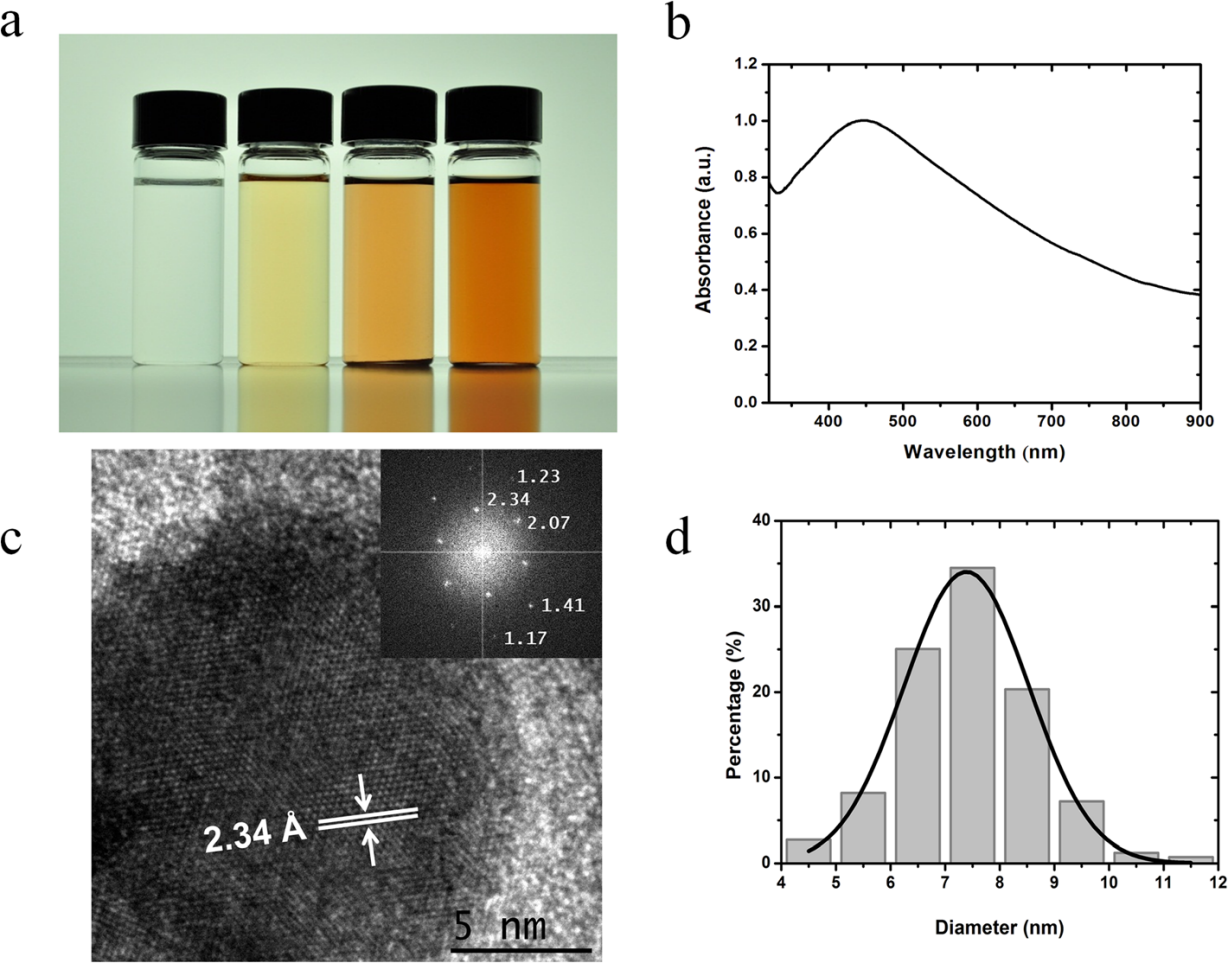
**Synthesis of silver nanoparticles in the cylindrical ethylene glycol micelles of the microemulsions. (a)** Visual appearance of the microemulsion. **(b)**. UV-vis spectrum of the sample **(c)** High-resolution electron microscopy micrograph of a nanoparticle. **(d)** Size histogram.

The silver nanoparticles were synthesized in the cylindrical ethylene glycol micelles of the microemulsions. When silver nitrate was added to the samples, a change in color was macroscopically evident in a time scale of hours. The initially transparent liquid became yellowish, then brownish, and eventually darkened. In Figure 2a, we show a picture displaying this visual time evolution, before (left) and after the beginning of the reduction reaction. The change in color is an indication that a reduction reaction has taken place and that silver nanoparticles have been formed. UV-vis spectroscopy results confirm this conclusion. In Figure 2b, an absorption spectrum has been measured of a sample after 15 days of AgNO_3_ addition. The curve displays a typical peak at 446 nm, associated with the plasmon absorption of silver nanocrystals [23]. We have confirmed that the particles are indeed silver nanocrystals by analyzing high-resolution transmission electron microscopy (HR-TEM) images. In Figure 2c, we display a typical HR-TEM micrograph where the atomic planes are clearly visible. The measured interatomic distances 2.34, 2.07, 1.41, 1.23, and 1.17 Å correspond to the planes (111), (200), (220), (311), and (222) of an fcc silver crystal (JCPDS file no. 4-0783). The inset shown in Figure 2c is a fast Fourier transform (FFT) pattern of the nanocrystal. Energy-dispersive X-ray spectroscopy (EDS) experiments confirmed the presence of silver in the arrays (in the Additional file 1: Figure S3 and Table S1). The obtained nanoparticles are mainly spherical and their mean diameter is **7** ± 1 nm; the statistical analysis was done about of 400 particles. The nanocrystals are relatively monodisperse (see histogram in Figure 2d); the experimental polydispersity index is *ε* = 0.1428.

**Figure 3:**
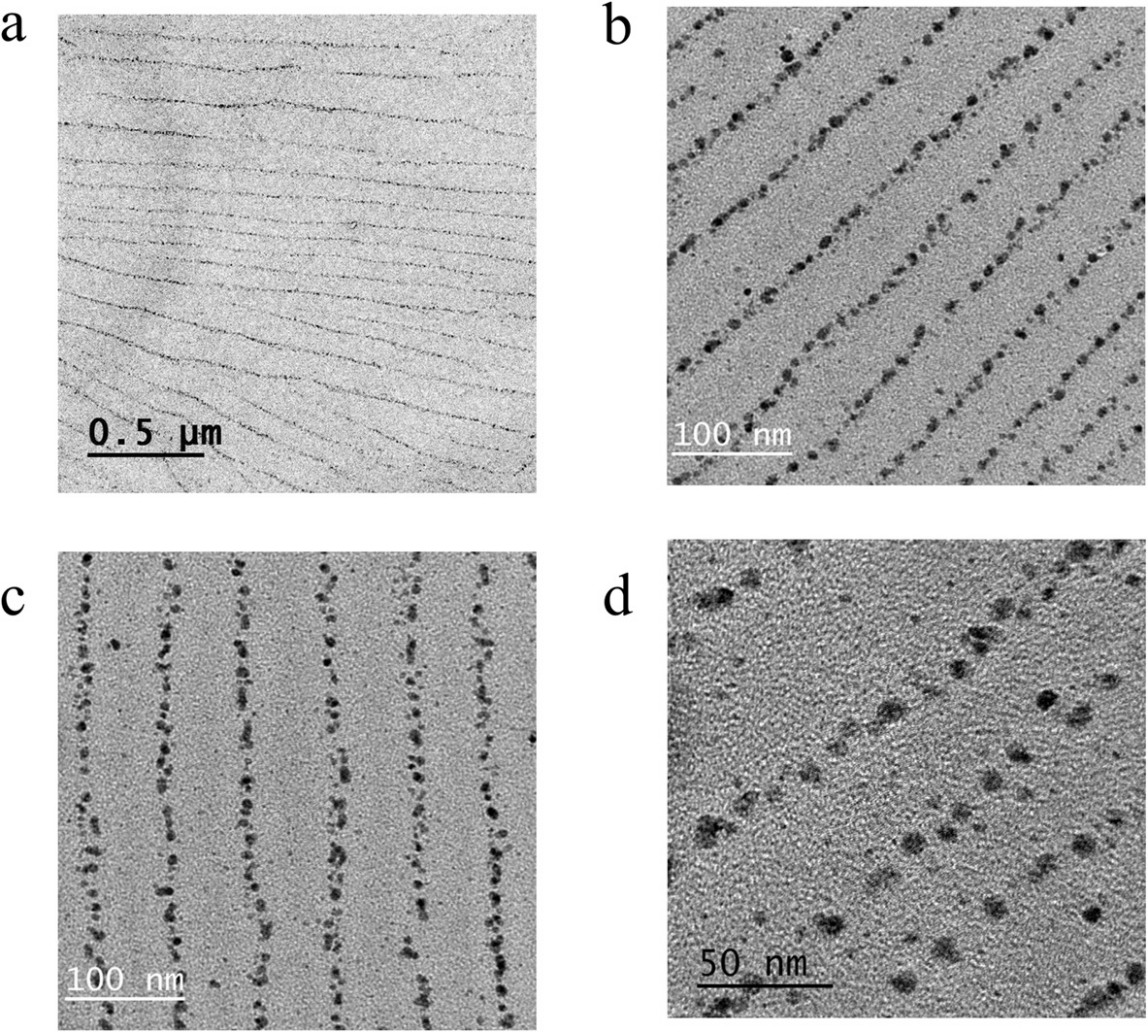
**Typical TEM images of the obtained nanoparticle arrays. (a)** Electron microscopy picture displaying the silver nanoparticle arrangement. **(b)**, **(c)** Micrograph with different views of the silver nanoparticle arrangement. **(d)** Zoom to the nanoparticle array.

When the nanoparticle-containing samples are deposited on a substrate and allowed to dry, the silver nanocrystals arrange in a manner expected from the microemulsion geometry. In Figure 3a,b,c,d, we present typical TEM images of the obtained nanoparticle arrays. The arrangements cover completely the field in the TEM pictures at low resolution (Figure 3a), spanning a surface of several square microns. Perhaps the most striking feature of Figure 3a,b,c,d is the clear ordering of the particles in highly parallel linear nanostructures. These alignments are as long as several microns (Figure 3a); the particles are confined in each line in a diameter of roughly 22 nm (Figure 3b,c). The average periodicity (center to center distance) of these lines is 70 nm. The linear density of nanoparticles along a stripe is of the order of 87 ± 11 particles/μm. In Figure 3d, we show a zoom on one of the lines; the individual silver nanoparticles are clearly distinguishable. We show more nanoparticle arrangements in Additional file 1: Figures S4 and S5.

The observed arrays are due to the microscopic structure of the microemulsion phase where the nanocrystals have been grown and to the deposition-drying process. The chosen, high-conductivity microemulsion where the synthesis takes place is composed of a network of long cylinders, which explains the high value of conductivity *κ*. As the reduction reaction takes place in the confined space defined by these channels, the resulting nanoparticles do not randomly disperse in the volume of the sample, but instead they remain aligned following the geometry of the micelles. When the samples are deposited on a substrate, the nanoparticles follow the ordering the microemulsion acquires. As isooctane evaporates, the ethylene glycol-rich cylinders pack in a hexagonal structure, with the particles in the core of the aggregates (Figure 1). Thus, the order observed in Figure 3a,b,c,d is reminiscent of the order in the microemulsion structure. This conclusion is confirmed by an estimation of the lattice distance *a*, in terms of the micelle volume fraction ϕ_mic_ in a hexagonal array. On geometrical grounds, it is straightforward to show that ${\phi}_{mic}=\frac{\pi }{2\sqrt{3}}{\left(\frac{d}{a}\right)}^2$, where *d* is the micelle diameter (it corresponds roughly to the diameter of the nanoparticle lines). If we evaluate the lattice distance from this expression using the experimental value for *d* and ϕ_mic_, we find *a* = 63 nm. The agreement is reasonably good with the experimental value *a* = 70 nm.

Note that similar linear nanostructures are obtaained in other adjacent regions of the microemulsion phase diagram (in the Additional file 1: Figures S1 and S4), confirming that two elements are required to obtain this ordering: to perform the reaction in a cylindrical micellar phase and to deposit/dry the sample in such a way that it packs in a hexagonal geometry.

**Figure 4:**
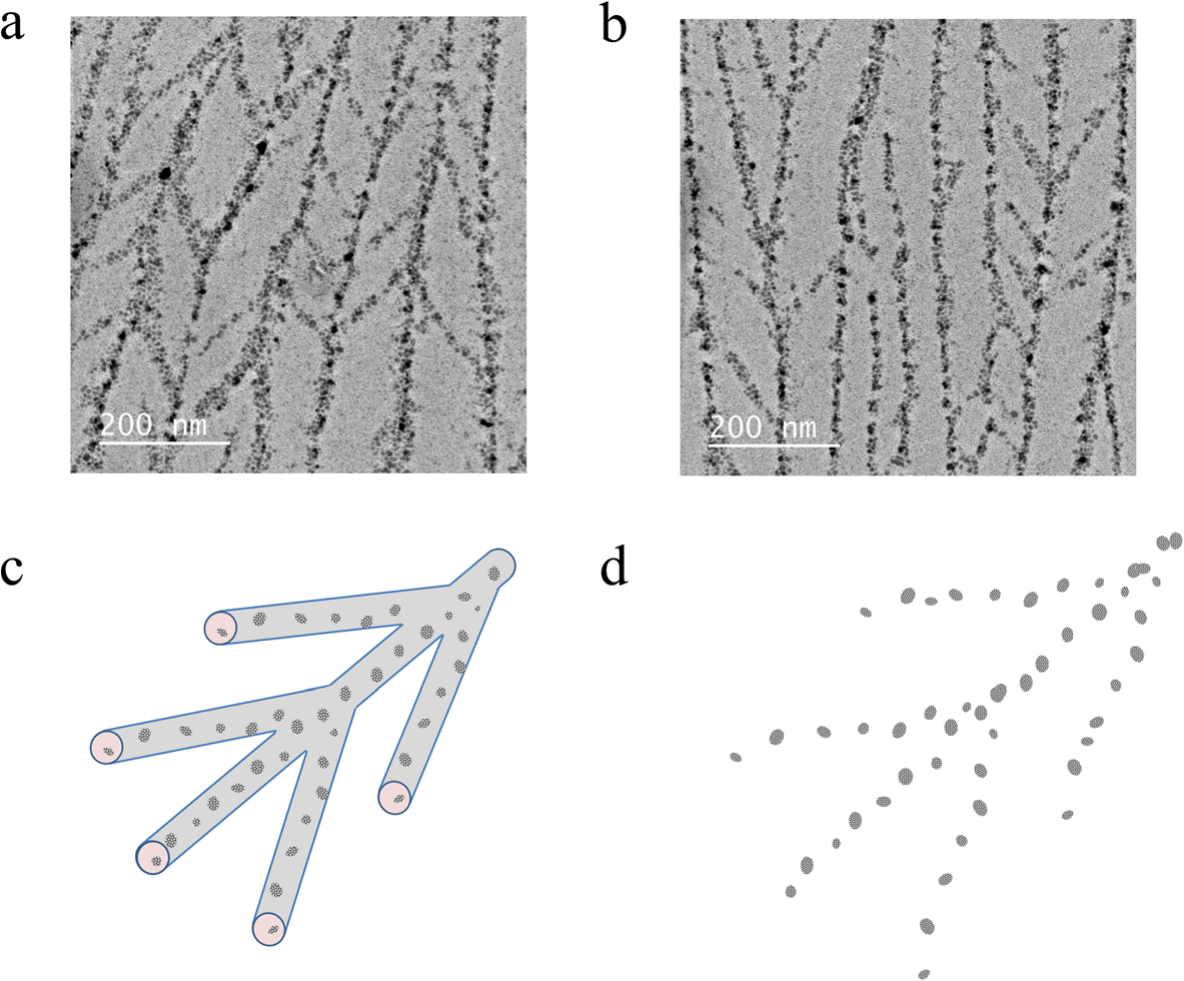
**TEM images of branched nanoparticle patterns. (a)**, **(b)** Two electron microscopy views of the branched patterns observed in the samples. **(c)** Depiction of a branched cylindrical micelle. **(d)** Expected pattern of the nanoparticle array.

The obtained nanoparticle patterns are composed of parallel lines, but in some cases, there is some degree of branching and interconnectivity as shown in Figure 4a,b. We have observed these branched arrays when the particles were synthesized with a threefold increased silver nitrate concentration. The average angle between a nanoparticle line and its branches is 36°. The branches connect adjacent cylinders. We attribute this result to some degree of connections in the array of cylindrical micelles where the reaction takes place, as depicted in Figure 4c. The connections are presumably induced by the higher AgNO_3_ concentration. Thus, the observed order in these nanoparticle arrays is reminiscent of this geometry (Figure 4d). We present more branched arrays in Additional file 1: Figure S6.

The method described in this paper allows both the synthesis of silver nanoparticles and their arrangement in well-defined linear patterns. The experiments are easy to perform and could allow the study of other processes relevant for nanoparticle science and engineering, such as particle coalescence or morphogenesis. In fact, in some cases, the nanocrystals are so close that coalescence is observed and leads to the formation of extended, fractal-like structures. On the other hand, the properties (optical, electrical) of the obtained arrays could be useful in applications such as photonics. For instance, the macroscopic size of the alignment length allows the light diffraction (results not shown).

**Figure 5:**
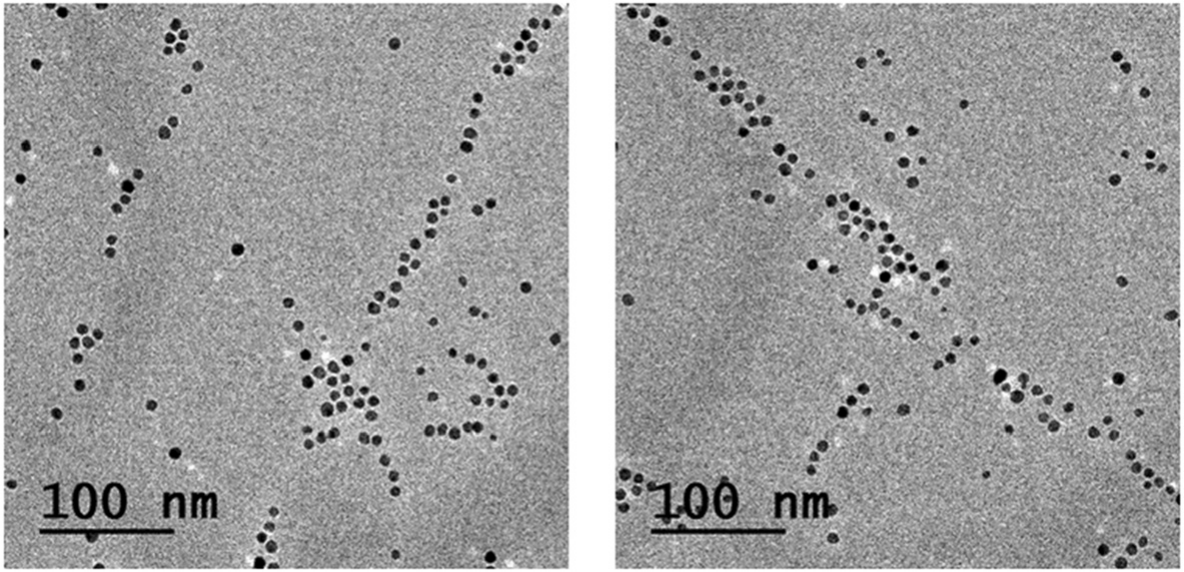
**Alignment of palladium nanoparticles obtained with the microemulsion method** (**preliminary results)** **.**

The method provides an easy way to obtain ordered nanostructures composed of silver nanoparticles. However, nanoparticles of other materials could be synthesized and ordered in the same way if other metal ions are reduced. We have proven the success of this strategy by aligning palladium nanocrystals synthesized with the microemulsion method; linear arrangements of Pd nanoparticles are observed in the preliminary experiments (Figure 5). We show some preliminary TEM pictures displaying arrays of palladium nanoparticles synthesized with this method. At this stage of the research, we are not sure why these arrays are not as regular as those obtained for silver. More experiments are underway in order to understand this point. Also, slight variations of the method could lead to versatile ways of obtaining nanoarrays. For instance, one can anticipate that the same arrangements can be obtained by injecting preformed nanoparticles in the microemulsion (in the Additional file 1: Figure S7a and b). That is, it is not a requirement to synthesize the particles *in situ*. This opens a wide range of possibilities of deposition of different materials. Furthermore, the method described in this paper could also allow the preparation of superimposed arrays of nanoparticles of one or several materials. One could get this kind of arrangements by depositing the sample in two or more steps in such a way that the micelles in each deposition have different orientations (in the Additional file 1: Figure S7c and d). The nanoparticles in each step can be of different materials.

## Conclusions

The non-aqueous microemulsion system obtained by the autoassembly of anionic surfactant (AOT), non-polar solvent isooctane, and polar solvent ethylene glycol promote the formation of inverses microemulsions; the non-polar solvent is in the highest proportion. Therefore, the polar solvent (EG) is within the micelle and in this place is performed the synthesis of silver nanoparticles. The isooctane is evaporated to room temperature, this process happens when the microemulsion is deposited on a substrate, and this generates a hexagonal phase that we have confirmed by polarizing optical microscopy. This hexagonal phase allows the self-alignment of the silver nanoparticles, with the formation of periodic arrangement, the separation of the silver nanoparticles between them is about 5 nm, and the separation distance between parallel and periodic lines is 70 nm. The mean size of the nanoparticles is 7 nm and the shape is quasi-spherical. These nanostructures would be very useful in applications where the electrical, magnetic, or optical properties need to be controlled or where some anisotropy in chemical composition is required.

## Additional file

Additional file 1:
**Supporting information.** Triangular phase diagram of the microemulsion system **(Figure S1)**. Electrical conductivity of the studied microemulsions **(Figure S2)**. Typical polarizing optical microscopy pictures of the microemulsion samples **(Figure S3)**. Additional silver nanoparticle arrays. The AgNO_3_ concentration is 20 mM **(Figure S4)**. Additional silver nanoparticle arrays. The AgNO_3_ concentration is 60 mM **(Figure S5)**. Typical EDS spectrum obtained for an arrangement of silver nanoparticles **(Figure S6)**. Chemical analysis of the EDS results for a silver nanoparticle **(Table S1)**. A wide range of possibilities of deposition of different materials **(Figure S7)**.
