# Supplementary material for: Self-alignment of silver nanoparticles in highly ordered 2D arrays
Source: Nanoscale Res Lett. 2015 Mar 1;10:101. doi: 10.1186/s11671-015-0804-8 (PMC4393402; doi:10.1186/s11671-015-0804-8)
Supplement: Additional file 1: — Supporting information. Triangular phase diagram of the microemulsion system (Figure S1). Electrical conductivity of the studied microemulsions (Figure S2). Typical polarizing optical microscopy pictures of the microemulsion samples (Figure S3). Additional silver nanoparticle arrays. The AgNO3 concentration is 20 mM (Figure S4). Additional silver nanoparticle arrays. The AgNO3 concentration is 60 mM (Figure S5). Typical EDS spectrum obtained for an arrangement of silver nanoparticles (Figure S6). Chemical analysis of the EDS results for a silver nanoparticle (Table S1). A wide range of possibilities of deposition of different materials (Figure S7). [file 11671_2015_804_MOESM1_ESM.pdf]

Additional File 1: Self Alignment of Silver Nanoparticles Highly Ordered in 2D Arrays

Rodríguez León E. et. al.

Microemulsion phase diagram

The microemulsions used as nanoreactors were prepared in the isooctane-rich corner of the microemulsion phase diagram. We have synthesized the nanoparticles along a dilution line (points 3 – 9 in figure S1) where the electrical conductivity has an increasing value. This variation indicates a shape transformation from spherical to cylindrical aggregates. In Figure S2 we plot the electrical conductivity of the microemulsions as a function of the ethylene glycol / AOT molar ratio (W), in the studied region. We have prepared the silver nanoparticles in these high-conductivity phases.

The alignments of silver nanoparticles reported in the manuscript were obtained in the point labeled 4 in Figure 1. However, similar alignments were obtained in several other neighboring points in the phase diagram (points labeled 3, 7, 9 and 20 in Figure S1). For instance, in figure S3 we display some TEM pictures of the nanoparticle arrays in these microemulsion compositions; we also show polarizing optical microscopy pictures of these samples. These results show that the ordering mechanism is the same as that reported in the paper: the packing of the cylindrical micelles in hexagonal geometries.

The microemulsion composition where we have performed most of the nanoparticle synthesis is point 4 in figure S1. The TEM pictures shown in the main manuscript are taken in this point. In Figures S4 and S5 we show more TEM micrographs of the alignments obtained in this point. The difference between these two sets of figures is the $AgNO_3$ concentration: 20 mM for Figure S4 and 60 mM for Figure S5.

The obtained silver nanoparticles and nanoarrays have been analyzed with TEM experiments. In the main manuscript we present TEM micrographs, as well as High-resolution TEM pictures and Fourier Processing using the software Digital Micrograph for the determination of the crystalline structure of the HR TEM images of the nanocrystals. All experiments agree with fcc silver crystals. In addition, we have performed Energy Dispersive X-ray microanalysis (EDS) experiments. They also confirm the presence of silver nanoparticles. In Figure S6 we present a typical EDS spectrum. The peaks corresponding to silver are clearly displayed. In addition, there are peaks related to Na and S, meaning that some surfactant (AOT) is present in the TEM grids. The Cu peak is due to the copper TEM grid. In Table S1 we present the chemical composition obtained from a typical EDS spectrum. Note that the silver/sodium and silver/sulfur molar ratios are consistent with the experimental molar $AgNO_3$/surfactant ratios.

These experiments suggest that more materials can be synthesized and aligned as shown for silver in the main text. We can even anticipate that the same arrangements can be obtained by injecting preformed nanoparticles in the microemulsion. In Figures S7a and S7b we

depict such a process. Finally, our method could also allow the preparation of superimposed arrays of nanoparticles of one or several materials. One could get this kind of arrangements by depositing the sample in two or more steps (Figures S7c and S7d).

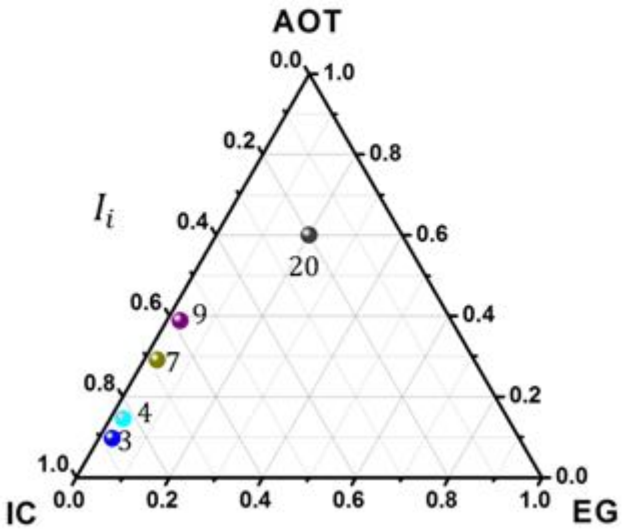

Figure S1. Triangular phase diagram of the microemulsion system, displaying the composition (weight proportion) of the studied samples. The samples are composed of isooctane (IC), ethylene glycol (EG) and surfactant (AOT). We have prepared silver nanoparticles in the points shown in this figure.

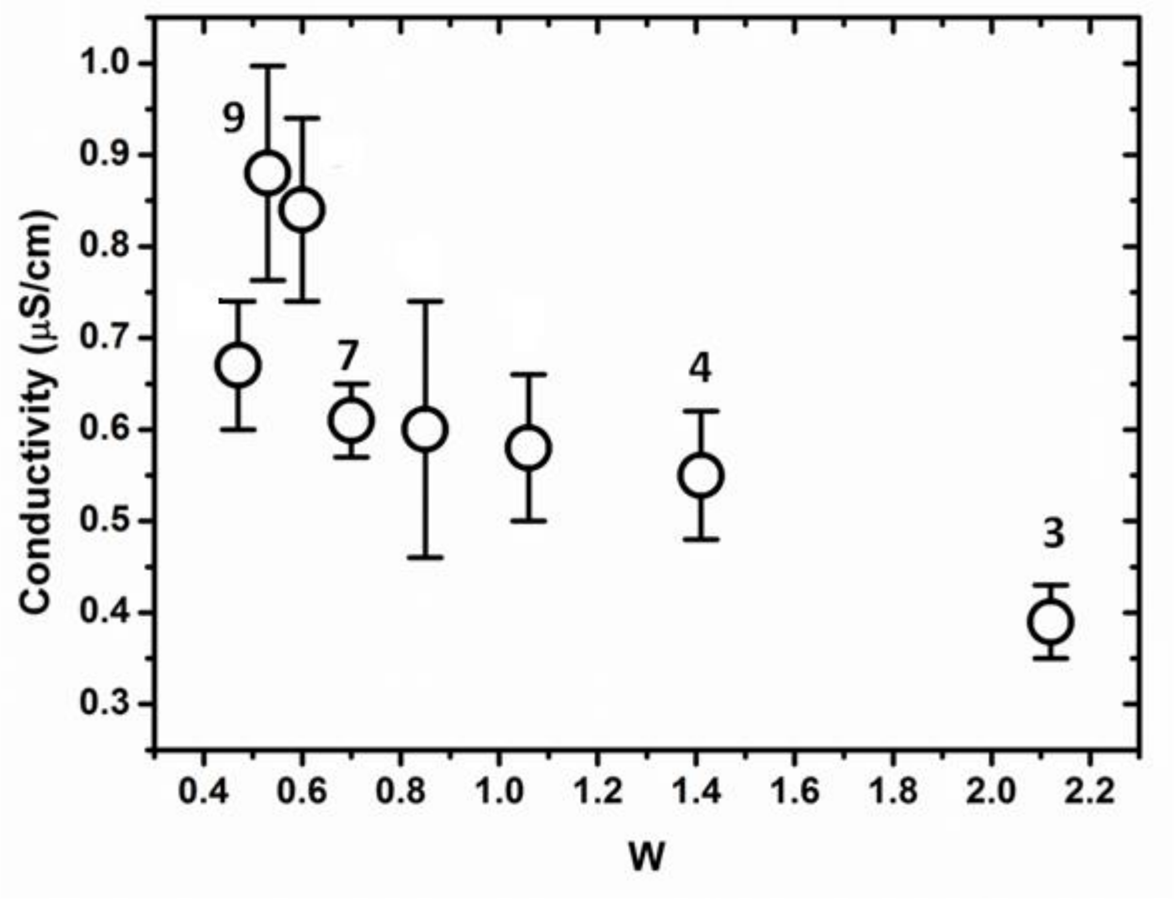

Figure S2. Electrical conductivity of the studied microemulsions as a function of W, the ethylene glycol / AOT molar ratio. The surfactant concentration increases from right to left. The labels correspond to the points of figure 1S.

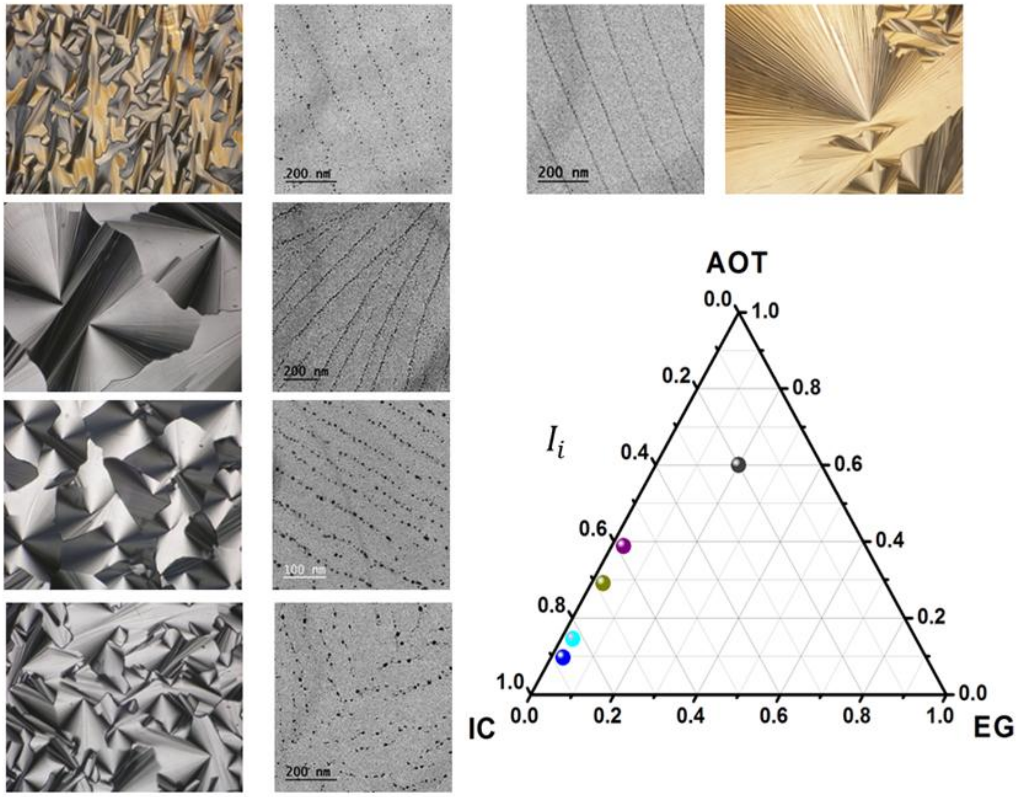

Figure S3. Typical polarizing optical microscopy pictures of the microemulsion samples, as well as TEM micrographs of the nanoparticle arrays obtained for the points of Figure 1. The numbers correspond to the points in Figures 1S and 2S.

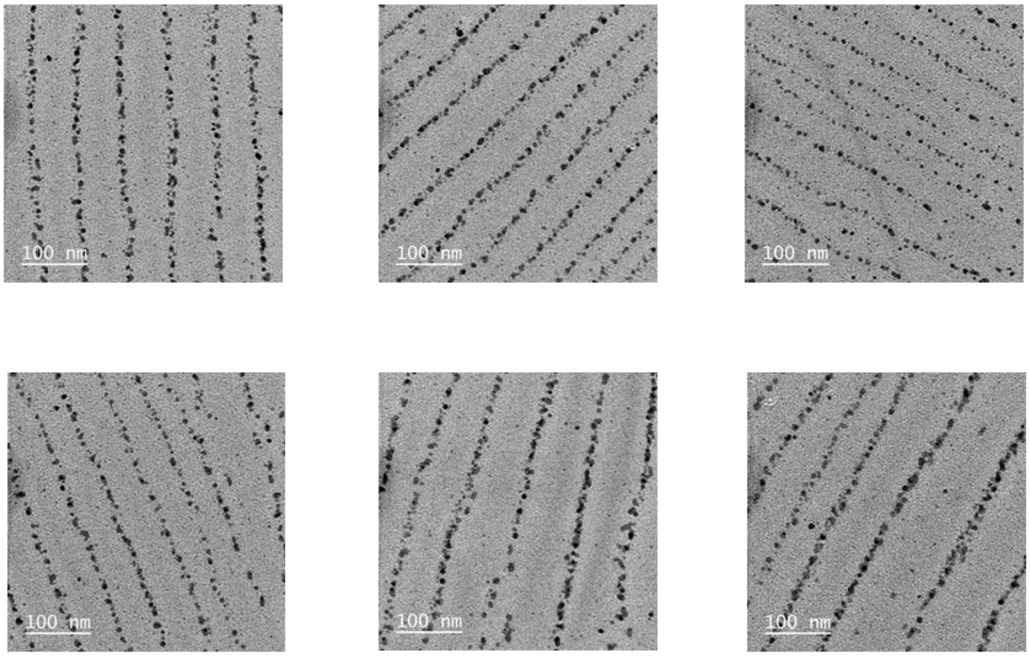

Figure S4. Additional silver nanoparticle arrays obtained performing the synthesis in point 4 of the phase diagram. The $AgNO_3$ concentration is 20 mM. The particles align along parallel lines.

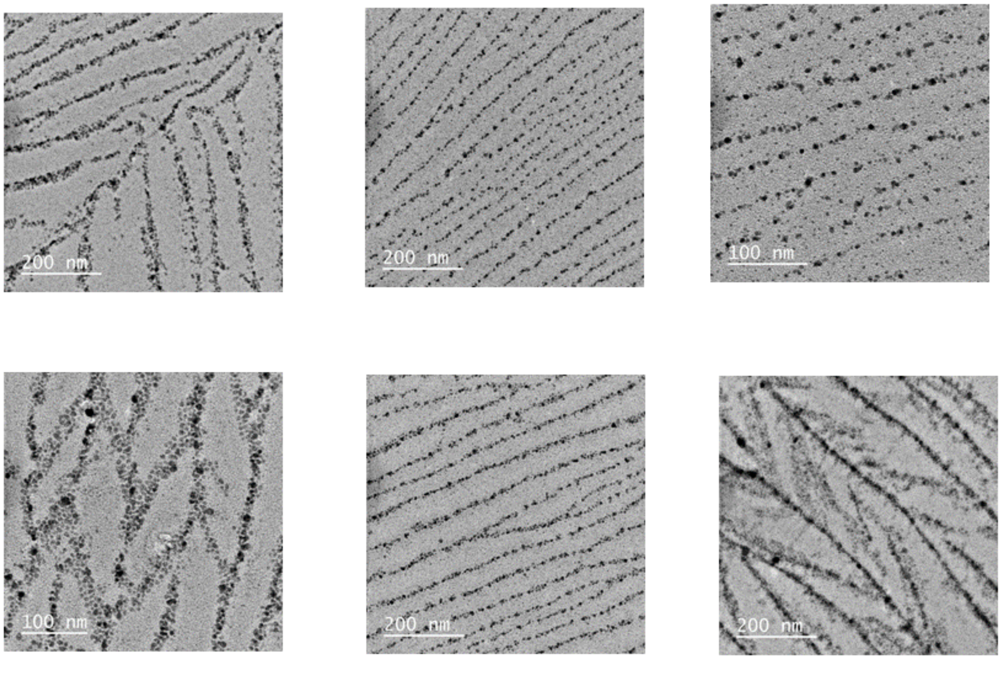

Figure S5. Additional silver nanoparticle arrays obtained performing the synthesis in point 4 of the phase diagram. The $AgNO_3$ concentration is 60 mM. Note that parallel as well as branched alignments are observed. The difference with figure 4S may be due to the different $AgNO_3$ concentration.

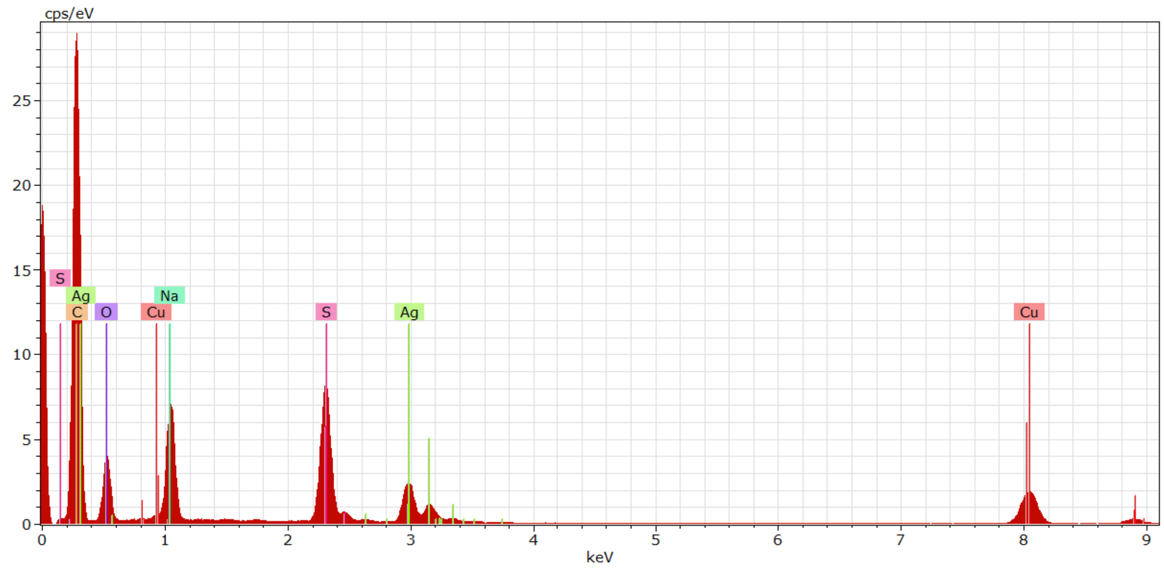

Figure S6. Typical EDS spectrum obtained for an arrangement of silver nanoparticles. The peaks of silver are clearly visible (Ag). The S and Na peaks are due to surfactant (AOT). The Cu peaks are due to the TEM grid.

Element	Atomic Number	X-ray Emission line	Element wt. %	Error in wt.%	Molar %
Oxygen	8	K-series	19.23002	0.621771486	35.9800987
Sulfur	16	K-series	31.59534	0.983571504	29.49610278
Sodium	11	K-series	20.37558	0.646324137	26.53149256
Silver	47	K-series	28.79906	1.081822791	7.992305954
Copper	29	K-series	0	0	0
Carbon	6	K-series	0	0	0
		Sum:	100		100

Table S1. Chemical analysis of the EDS results for a silver nanoparticle.

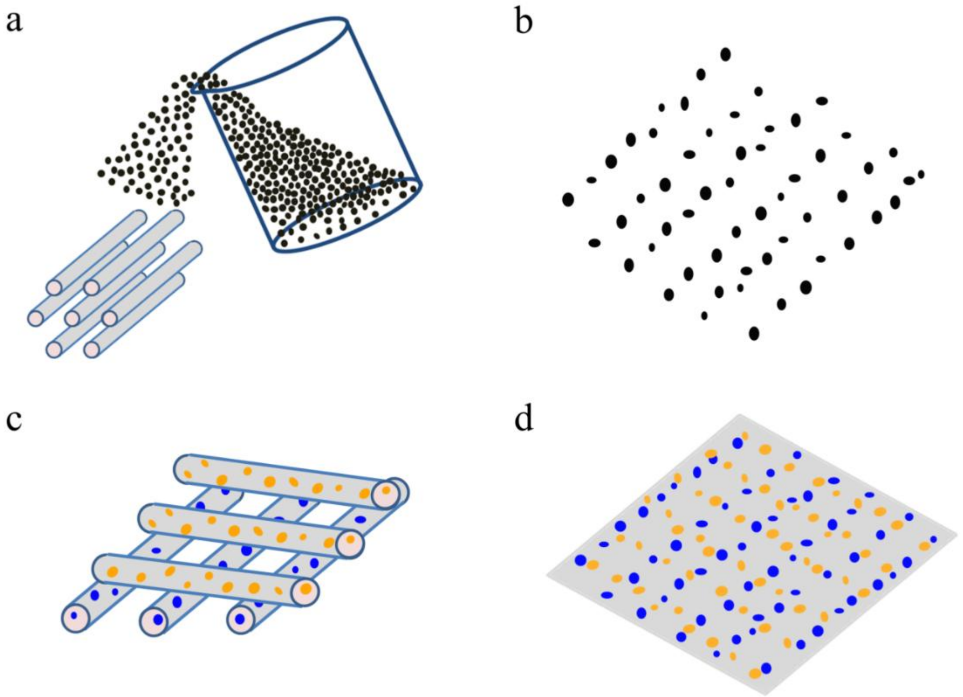

Figure S7. (a) Depiction of nanoparticles being poured into an "empty" microemulsion. (b) Expected nanoparticle array after deposition of the microemulsion on a substrate. (c) Depiction of the two-step deposition of microemulsions with different materials. (d) Anticipated array of nanoparticles of different materials deposited on a substrate with a two-step process.
